# Role of Berberine as a Potential Efflux Pump Inhibitor against MdfA from Escherichia coli: *In Vitro* and *In Silico* Studies

**DOI:** 10.1128/spectrum.03324-22

**Published:** 2023-02-14

**Authors:** Ying Li, Xizhen Ge

**Affiliations:** a College of Biochemical Engineering, Beijing Union University, Beijing, China; University of Exeter

**Keywords:** berberine, efflux pump, inhibitor, salt bridge, conformational transition

## Abstract

Infections by Gram-negative pathogens are usually difficult to manage due to the drug export by efflux pumps. With the evolution and horizontal transfer of efflux pumps, there is an urgent need to discover safe and effective efflux pump inhibitors. Here, we found that the natural compound berberine (BBR), a traditional medicine for intestinal infection, is an inhibitor against the major facilitator superfamily (MFS) efflux pump MdfA in Escherichia coli. The impact of BBR on MdfA was evaluated in a recombinant E. coli reporter strain. We demonstrated that low levels of BBR significantly increased intracellular ciprofloxacin concentrations and restored antibiotic susceptibility of the reporter strain. At the same time, we conducted molecular dynamics simulations to investigate the mechanisms of BBR’s effect on MdfA. Our data indicated that BBR can aggregate to the periplasmic and cytoplasmic sides of MdfA in both of its inward and outward conformations. Protein rigidities were affected to different degrees. More importantly, two major driving forces for the conformational transition, salt bridges and hydrophilic interactions with water, were changed by BBR’s aggregation to MdfA, which affected its conformational transition. In summary, our data provide evidence for the extended application of BBR as an efflux pump inhibitor at a clinically meaningful level. We also reveal the mechanisms and provide insights into BBR’s effect on the reciprocal motion of MdfA.

**IMPORTANCE** In this work, we evaluated the role of berberine (BBR) as an inhibitor of the MFS efflux pump MdfA from E. coli. We demonstrated that low levels of BBR significantly increased intracellular ciprofloxacin concentrations and restored antibiotic susceptibility of the reporter strain. Molecular dynamics simulations revealed the effect of BBR on the conformational transition of MdfA. Our data suggested that driving forces for MdfA’s conformational transition were affected by BBR and provided evidence for BBR’s extended application as an effective inhibitor of MdfA.

## INTRODUCTION

Infectious diseases are considered to be the primary leading causes of death around the world, and nearly 10% of them are caused by bacterial infections (1). However, overuse and misuse of antibiotics has resulted in an explosion of antibiotic resistance, which now is a public health issue of greatest importance (2). Among the factors that contribute to the survival of bacterial pathogens in the presence of antibiotics, efflux pumps play a crucial role by transporting a broad range of substrates out of the cytoplasm (3). Indeed, some previously effective antibiotics used for treating Gram-negative pathogen infections, such as azithromycin, streptomycin, and tetracycline, have become less effective because of efflux pumps (4).

Until now, six families of proton-driven efflux pumps have been identified in Gram-negative pathogens, namely, the resistance-nodulation-cell division (RND) family, multidrug and toxic compound extrusion (MATE) family, major facilitator superfamily (MFS), small multidrug resistance (SMR) family, proteobacterial antimicrobial compound efflux (PACE) family, and *p*-aminobenzoyl-glutamate transporter (AbgT) family (5–7). Among these efflux pumps, the MFS efflux pump family has been deduced to contain the largest and most diverse family among all the efflux pump superfamilies in all kingdoms of life (8). For instance, 25% of prokaryotic transporters belong to the MFS family, and there are more than 100 MFS efflux pumps in the human genome (9, 10). In Gram-negative pathogens, some representative MFS efflux pumps have been investigated, such as MdfA from Escherichia coli, KmrA from Klebsiella pneumoniae, and SmvA from Salmonella enterica (11–13). Studies of antibiotic susceptibilities indicate that these MFS efflux pumps often confer a narrow range of quinolone resistance, which is an important class of drug in antibiotic treatment of severe infections (14). More seriously, increasing evidence suggests that these efflux pumps are being transferred via mobile genetic elements, posing potential concerns for antibiotic therapy clinically (15, 16). Therefore, discovery efforts for effective and safe inhibitors continue.

**FIG 1:**
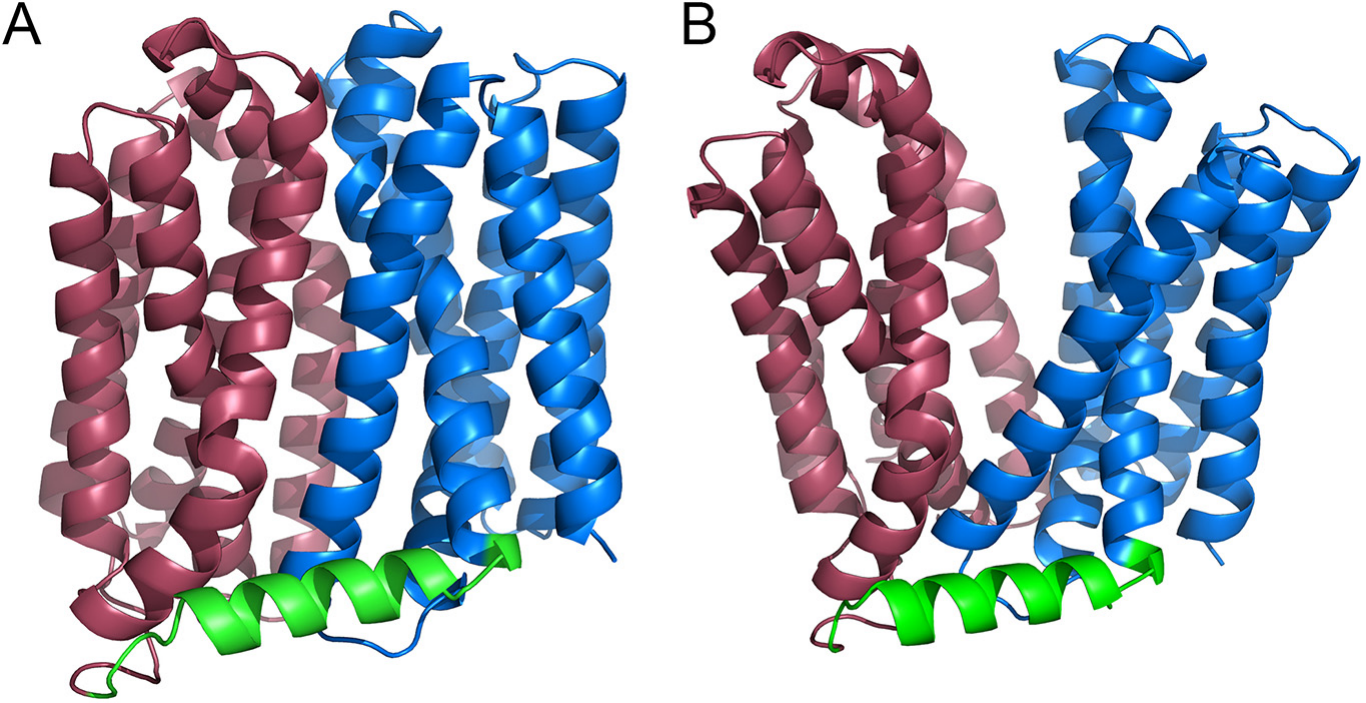
Structures of MdfA^inward^ (A) and MdfA^outward^ (B). The N-repeat, C-repeat, and their connecting hinge loop are colored with brown, blue, and green, respectively. Visualization of the structure was conducted in PyMOL.

The MFS efflux pump is embedded in the inner membrane of Gram-negative pathogens (17). The structure of an MFS efflux pump is composed of two six-helix rigid domains forming a central transmembrane channel (8, 18), and a rocker-switch model has been proposed to describe the export process by switching between inward- and outward-facing conformations (Fig. 1), which are believed to be the excited and ground states, respectively (19). Based on the mechanism of reciprocal movement, two kinds of inhibitors have been investigated to reduce drug export. First, ionophores such as carbonyl cyanide *m*-chlorophenyl hydrazone (CCCP) are used to disrupt the proton motive force and thereby inactivate all importers and exporters, including MFS efflux pumps (20). However, these inhibitors are usually toxic to humans. The other kind of inhibitor is MFS specific, such as verapamil and omeprazole, while the high concentrations at which efflux pumps are inhibited (usually over 50 μg/mL) also limit their applications (21).

Plant-derived secondary metabolites are believed to be the main source of natural efflux pump inhibitors (22). Among the identified isoquinoline alkaloid compounds, berberine (BBR) is a promising one due to its safety for humans. Indeed, BBR has been widely used for the treatment of intestinal infections, as it blocks the adherence of pathogens to the inner surface of the intestine (23). Moreover, the relationship between BBR and efflux pumps has also been illustrated. First, as a fluorescent compound which can be excited at 335 nm, BBR is often applied to monitor the efflux behavior of bacteria (24). Second, BBR has been demonstrated to hijack the MFS efflux pump Mdr1p and restore antibiotic susceptibility of Candida albicans, thus showing great potential as a safe efflux pump inhibitor (25). However, the inhibitory mechanisms behind this action are still unclear. First, among the characterized structures of MFS efflux pumps, only a few of them are in outward-facing conformations (ground state). The lack of different outward-facing conformations of MFS efflux pumps has restricted our understanding of the ground state (26). Moreover, export of substrate is accomplished by a large conformational transition of an efflux pump, and substrates will interact with different regions or motifs during the whole cycle. Therefore, molecular docking is insufficient to describe this dynamic process. To better understand the interactions between BBR and MFS efflux pumps, more investigations should be conducted.

Measuring antibiotic efflux in Gram-negative pathogens is an important way to estimate the level of antibiotic resistance (27). Bacterial efflux can be evaluated at either the population level or single-cell level (28). Conventional determinations of efflux at the population level can be divided into direct measurement of efflux with ethidium bromide and measurement of intracellular accumulation of an efflux substrate (28, 29). These methods have bee demonstrated to be useful in estimating the effects of an inhibitor on certain efflux pumps (30, 31). With the development of chemical biology and microfluidics equipment, efflux in a single-cell or cell-free system can be applied to detect or visualize efflux with modified fluorescent substances (32–34). For MFS efflux pumps from Gram-negative pathogens, their roles and substrates have been clearly elucidated. These efflux pumps usually confer a narrow range of antibiotic resistance against quinolones (35). Therefore, measuring bacterial sensitivity and intracellular substrate concentration are supposed to evaluate the efficiency of inhibitor against the overexpressed efflux pump.

Here, we apply a representative MFS efflux pump, MdfA, the two conformations of which have been captured, to study BBR’s inhibitory effect *in vitro* and *in silico*, and the commercially available pure BBR compound was used. We constructed a recombinant E. coli strain to test its antibiotic susceptibility and intracellular antibiotic concentrations in the presence of BBR. At the same time, conventional molecular dynamics simulations were carried out to demonstrate the impact of BBR on the conformational transition of MdfA. We aimed to provide new insights into the interactions between BBR and MdfA.

## RESULTS

### BBR inhibited the activity of MdfA in the reporter strain.

**FIG 2:**
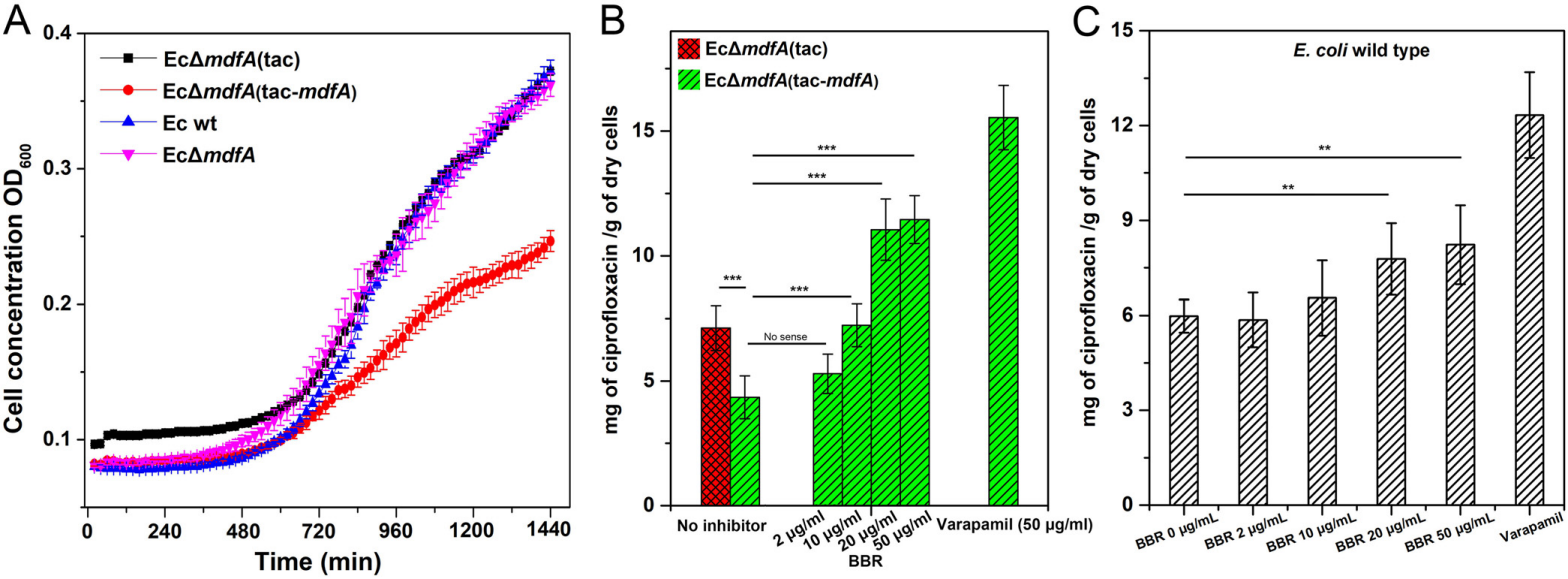
Cell growth and intracellular ciprofloxacin concentrations of the reporter strain. (A) Overexpression of *mdfA* resulted in decreased cell growth. (B) Overexpression of *mdfA* conferred a reduced intracellular ciprofloxacin concentration for E. coli, while supply of BBR reversed it. (C) BBR increased the intracellular ciprofloxacin concentration of E. coli wild-type strain moderately. All the experiments were conducted with at least three biological replicates. Data are presented as mean values ± standard errors. ***, *P < *0.01; ***, *P < *0.001.

In order to identify the effect of BBR on the activity of MdfA, we constructed an E. coli reporter strain with deletion of the *mdfA* gene on the chromosome (EcΔ*mdfA*). Next, we overexpressed the *mdfA* gene in this strain with a plasmid derived from pET-28a, making an isopropyl-β-d-thiogalactopyranoside (IPTG)-inducible expression cassette, tac-*mdfA* [EcΔ*mdfA*(tac-*mdfA*)]. Then, growth curve analysis with the reporter strain was conducted (Fig. 2A). Compared to the wild-type E. coli, mutation of native *mdfA* had no impact on cell growth. However, when *mdfA* was overexpressed in E. coli, significantly decreased cell growth was observed. The final cell concentration of the recombinant strain was only 60% of that of the wild type, while no delayed cell growth was observed. Overexpression of efflux pump conferred reduced cell growth, a phenomenon also identified in other reports (36–38). Next, we investigated the effect of BBR on the cell growth of the reporter strain and the wild-type strain (see Fig. S1 and S2 in the supplemental material). The results indicated that cell growth was rarely affected by BBR.

Subsequently, we evaluated the MIC values of the reporter strain with gradient concentrations of BBR in the medium (Table 1), and several efflux-related antibiotics were used. Mutation of *mdfA* gene on the chromosome of E. coli resulted in a moderately decreased MIC only for ciprofloxacin, while the effectiveness of the other antibiotics was not changed. In contrast, overexpression of *mdfA* conferred a significantly elevated MIC for ciprofloxacin (8-fold), while the MICs of the other antibiotics were not affected to a meaningful level. The results indicated that MdfA conferred a narrow range of antibiotic resistance, and similar results have also been reported in the literature (14, 39). Next, we tested BBR’s effect on MICs of antibiotics against the reporter strain. With increased concentrations of BBR in the medium, MICs of ciprofloxacin were decreased sharply. When the concentration of BBR was 20 μg/mL, the MIC of ciprofloxacin decreased to 0.008 μg/mL, which indicated that BBR restored susceptibility of ciprofloxacin. Interestingly, MICs of the other antibiotics were also decreased at high BBR concentrations, indicating that synergies were detected between BBR and the second antibiotics. However, since overexpression of *mdfA* had no effect on promoting their MICs, we concluded that these synergies were not conferred by BBR’s impact on MdfA.

**TABLE 1:** MICs of efflux-related antibiotics against wild-type and recombinant strains

Antibiotic	MIC (μg/mL)							
	Ec wt	EcΔ*mdfA*	EcΔ*mdfA*-tac	EcΔ*mdfA*-tac-*mdfA* when BBR added to medium at:				
				0 μg/mL	2 μg/mL	10 μg/mL	20 μg/mL	50 μg/mL
Tetracycline	0.625	0.625	0.625	1.25	1.25	1.25	1.25	0.625
Azithromycin	0.625	0.3125	0.625	0.625	0.625	0.625	0.3125	0.3125
Ciprofloxacin	0.004	0.002	0.004	0.032	0.032	0.016	0.008	0.004
Rifampicin	12.5	12.5	12.5	12.5	12.5	12.5	6.25	6.25
Roxithromycin	0.125	0.125	0.125	0.125	0.125	0.125	0.125	0.0625
Streptomycin	0.75	0.75	0.75	0.75	0.75	0.75	0.375	0.375
Nalidixic acid	2	2	2	2	2	2	2	1
Ampicillin	1	1	1	1	1	0.5	0.5	0.25

Based on the results of antibiotic susceptibility testing, we further measured the intracellular concentrations of ciprofloxacin in the presence of gradient concentrations of BBR (Fig. 2B). Overexpression of *mdfA* resulted in a significantly decreased intracellular ciprofloxacin concentration, indicating MdfA can confer ciprofloxacin resistance. Next, gradient concentrations of BBR were added in the cultivation stage. As a result, 10 μg/mL of BBR effectively increased the intracellular ciprofloxacin concentration, and this was further promoted in the presence of 20 μg/mL BBR. However, when the concentration of BBR was increased to 50 μg/mL, intracellular ciprofloxacin was not further elevated. We also conducted the experiments with verapamil, which is an MFS-specific inhibitor, at 50 μg/mL. The results indicated that 20 μg/mL BBR had nearly 70% of effectiveness compared to 50 μg/mL of verapamil in increasing the intracellular ciprofloxacin concentration. We also measured the intracellular ciprofloxacin level in wild-type E. coli (Fig. 2C), and BBR functioned in a similar way compared to the data for the reporter strain, while the efficiency was much lower. Altogether, our data indicated that BBR can affect MdfA’s drug export and has a potential auxiliary role in antibiotic therapy.

### BBR affected total and local flexibilities of MdfA.

**FIG 3:**
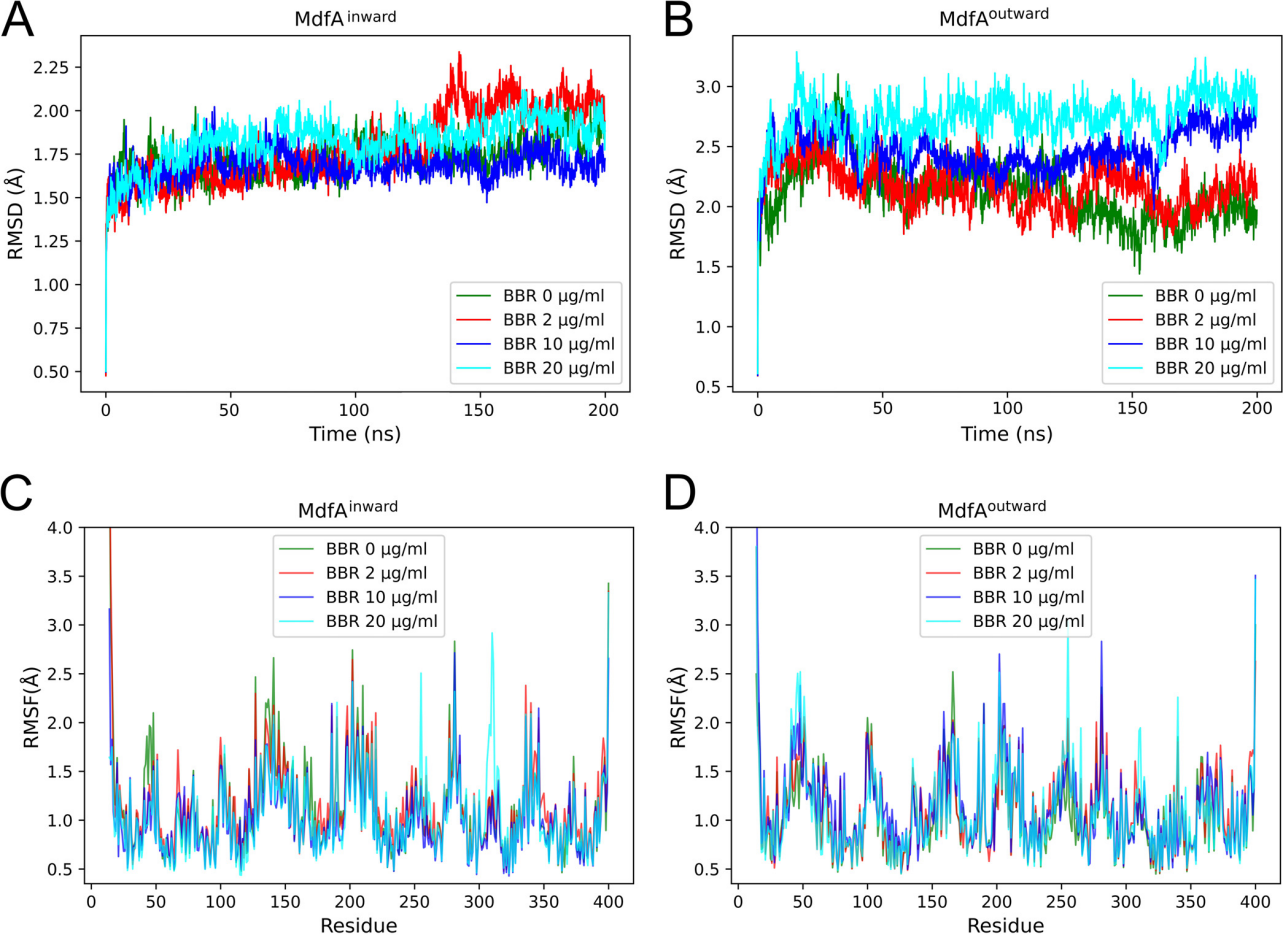
BBR affected total and local flexibilities of MdfA^inward^ and MdfA^outward^. The RMSD was used to evaluate the total flexibilities of MdfA (A and B) and the RMSF was applied to determine the detailed changes of each residue (C and D).

To explore the mechanisms of BBR’s impact on MdfA, we conducted molecular dynamics simulations of MdfA^inward^ and MdfA^outward^ in gradient concentrations of BBR. First, flexibilities of MdfA were calculated in all these simulations (Fig. 3). According to the root mean square deviation (RMSD) results, which indicated the total flexibility of the protein (Fig. 3A and B), BBR had no significant effect on the flexibility of MdfA^inward^, while that of MdfA^outward^ was elevated. Then, local flexibility was calculated as the root mean square fluctuation (RMSF) to evaluate the effect of BBR on the detailed changes of MdfA (Fig. 3C and D). As a result, flexibilities of regions Tyr255 to Tyr257 and of Thr306 to Tyr313 were elevated significantly in both MdfA^inward^ and MdfA^outward^. These regions were both located at the periplasmic side of the C-repeat. Conversely, for the region of the N-repeat at the periplasmic side (Gln46 to Asp52), flexibilities of MdfA^inward^ were decreased while those of MdfA^outward^ were promoted. Since the altered flexibilities were located at each side of MdfA, we deduced that the increased flexibilities were caused by MdfA’s interaction with BBR molecules, which were fluid in the simulation system.

### Hydrophobic interactions between BBR and MdfA.

**FIG 4:**
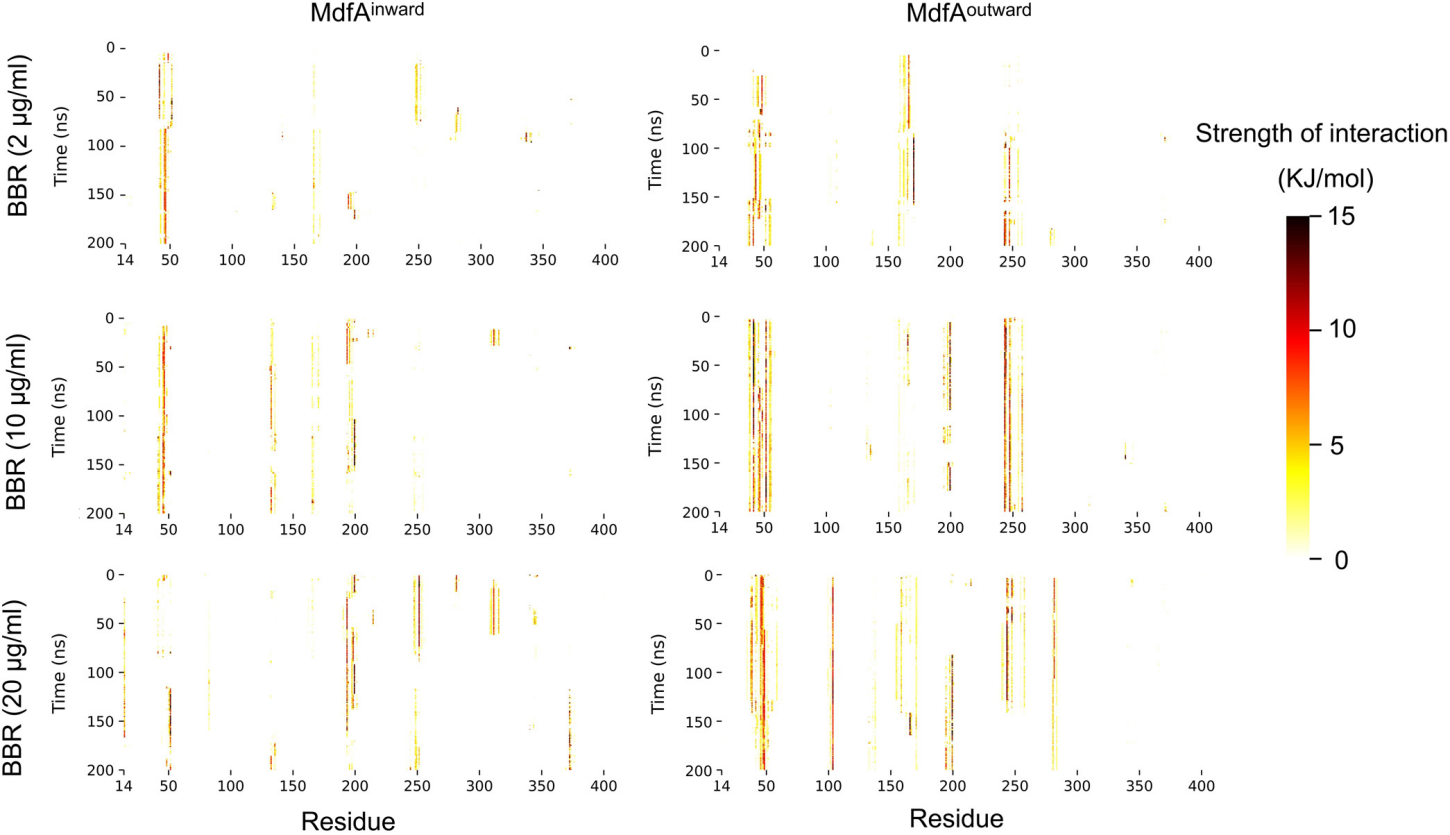
BBR aggregated to certain residues at both the periplasmic side and cytoplasmic side and formed stable interactions. For each residue of MdfA, the hydrophobic interaction strength with BBR molecules was calculated, and the interaction strength was visualized as a heatmap.

In our recent work, we demonstrated that as a hydrophobic molecule, BBR can inhibit the activity of cutinase by hydrophobic interactions with the catalytic center (40). Since MdfA’s hydrophilic interactions with water and hydrophobic interactions with membrane are important driving forces for its conformational transition (8), we calculated the effect of BBR on MdfA via hydrophobic interaction (Fig. 4 and Fig. S3). The results indicated that as the concentration of BBR increased, more residues were detected to have hydrophobic interactions with BBR at high levels. For MdfA^inward^ at 2 μg/mL of BBR, strong hydrophobic interactions were observed between BBR and the region from Leu41 to Val44, the region located at the periplasmic side of the N-repeat. As concentrations of BBR were increased, extended interactions were detected for regions of Glu132 to Glu135, Leu193 to Ile199, Ile247 to Gly249, and Trp372 to Gly376. Notably, two of the above regions were located at the cytoplasmic side of MdfA. Since the inward conformation reveals MdfA’s activation for accommodation of substrates, the extended interactions with BBR at the cytoplasmic side were deduced to have an effect on this process via steric hindrance.

For MdfA^outward^, a similar phenomenon was observed upon comparison to MdfA^inward^, while the regions that had interactions with BBR were altered (Fig. S3). First, stronger interactions were also found at Leu41 to Val44. Moreover, extended interactions were detected at three regions, namely, Leu101 to Asp103, Glu194 to Ile199, and Thr279 to Val284. Importantly, two of the regions were found to have interactions with BBR only at 20 μg/mL (Leu101 to Asp103 and Thr279 to Val284). MdfA’s activation depended on the protonation of key residues in the center of the helix channel (e.g., Asp34). As an alkalescence molecule, BBR’s impact on activation of MdfA was deduced by hindering the entrance of protons, since BBR was concentrated in the periplasmic side of MdfA^outward^.

### Salt bridges were decreased for MdfA^inward^ while increased for MdfA^outward^.

**FIG 5:**
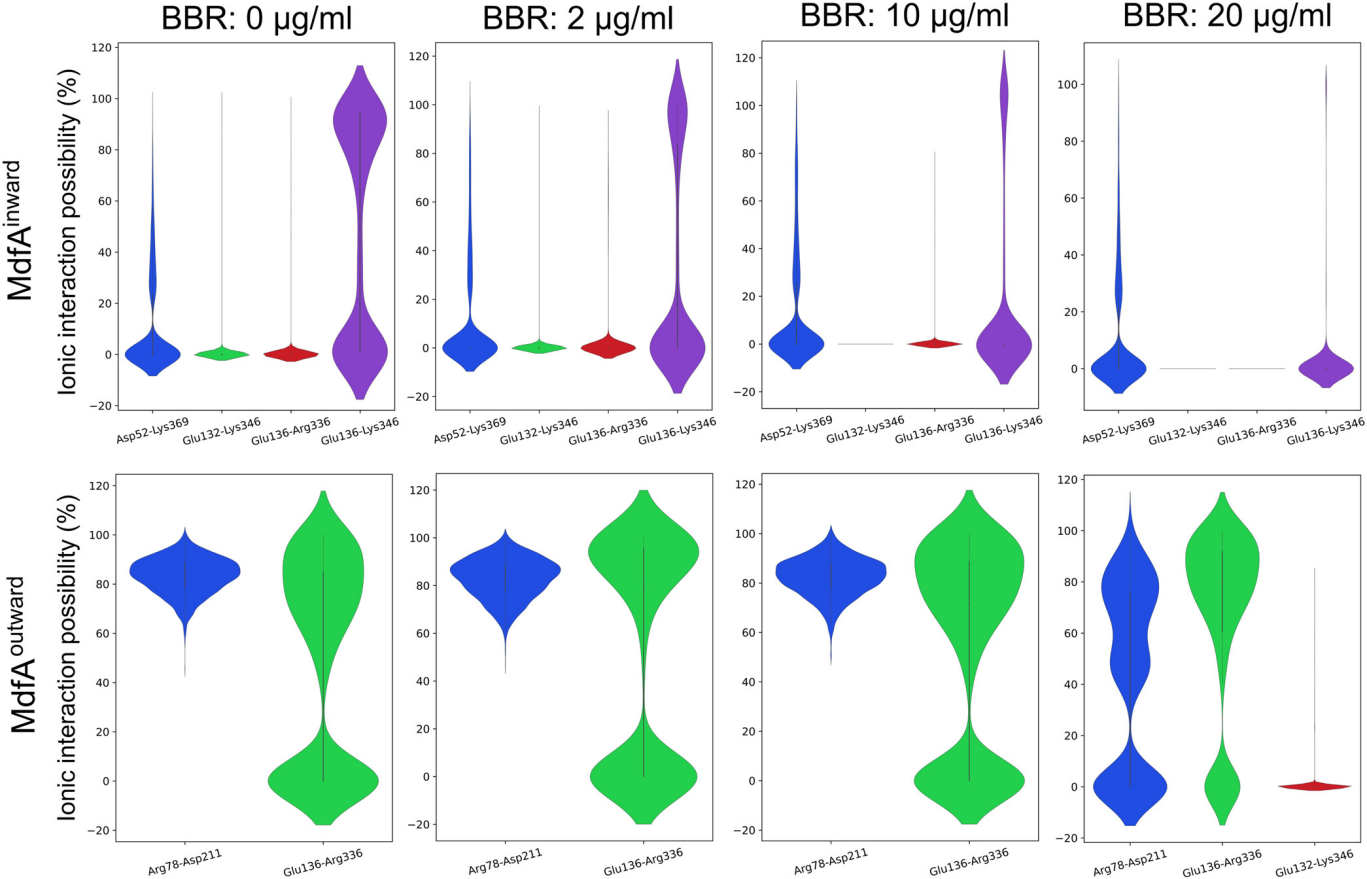
Supply of BBR decreased salt bridge interactions of MdfA^inward^, while if increased interactions of MdfA^outward^. Salt bridges were counted if the distance between the positively and negatively charged residues was lower than 5 Å. A violin plot was used to exhibit the possibility of each salt bridge.

**FIG 6:**
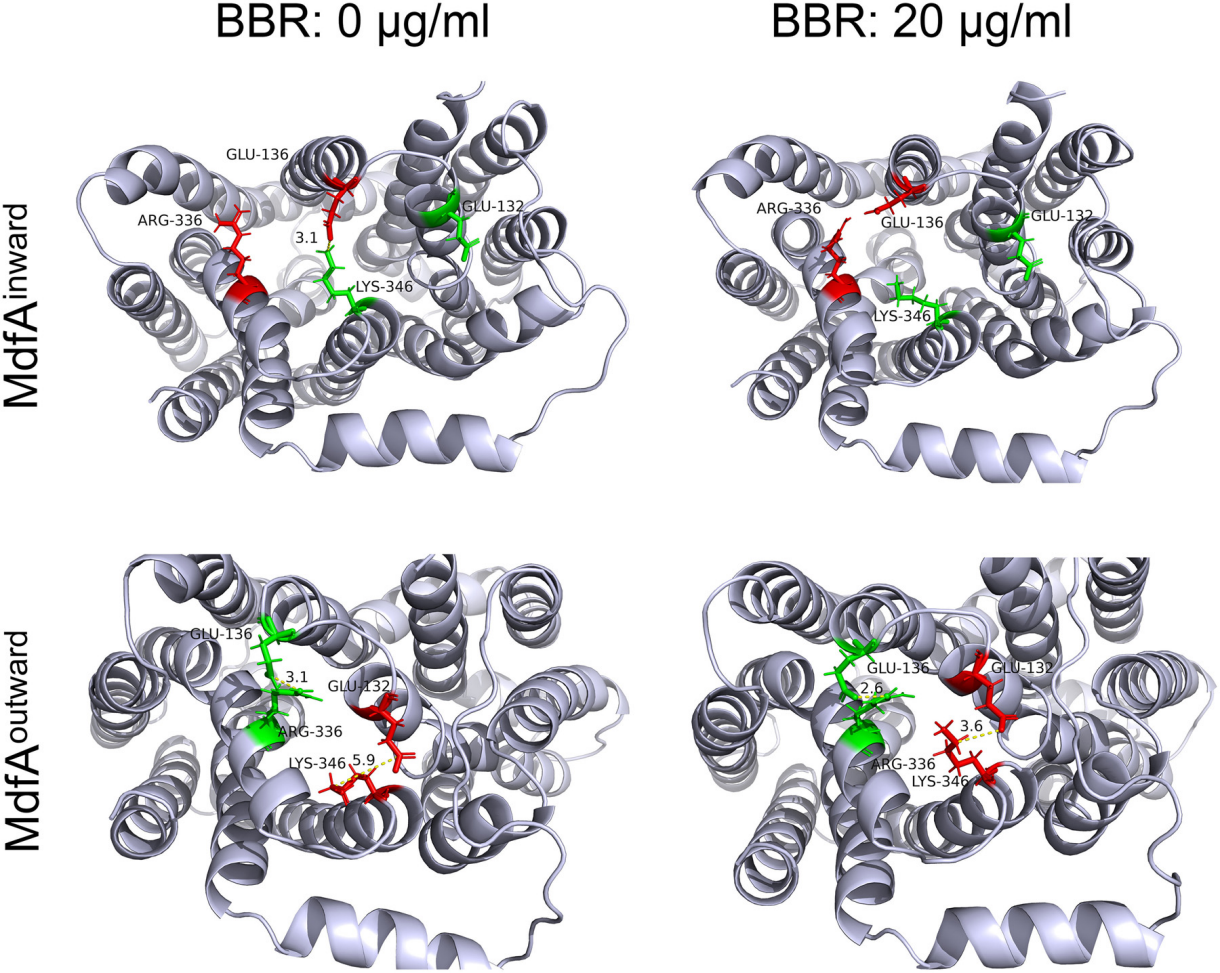
Visualization of salt bridges from cytoplasmic side of MdfA^inward^ and MdfA^outward^. Visualizations were conducted in PyMOL.

Salt bridges, which locate across the N- and C-repeats at both sides of MdfA, provide important driving forces for its conformational transition (8). Since BBR was determined to be aggregated at the two sides of MdfA, we calculated these salt bridges to investigate BBR’s impact on them (Fig. 5 and 6). For MdfA^inward^, four salt bridges were detected, and three of them were at the cytoplasmic side. Interestingly, BBR reduced salt bridge possibilities at the cytoplasmic side of MdfA, while at the periplasmic side, the region from Asp52 to Lys369 was not affected, even at the highest concentration of BBR. At cytoplasmic side, increased concentrations of BBR conferred sharply reduced salt bridge possibilities. At 20 μg/mL of BBR, two salt bridges were not formed throughout the simulation, and the other salt bridge, Glu136 to Lys346, also decreased to a much lower level. More importantly, no altered salt bridges were observed. In contrast, for MdfA^outward^, no differences were discovered at 2 and 10 μg/mL of BBR compared to the control, while increased strength of Glu136-Arg336 was detected at 20 μg/mL of BBR. Moreover, a new salt bridge, Glu132-Lys346, was discovered with low possibility, which was also at the cytoplasmic side of MdfA. These two salt bridges might increase the difficulty for MdfA’s activation, since they are located across the entrance of the binding pocket. Conversely, another salt bridge, Arg78-Asp211, of the cytoplasmic side was reduced at the highest concentration of BBR. Asp211 located near the connecting hinge loop between the N- and C-repeats, and this alteration was deduced to be affected by the increased interaction of Glu136-Arg336 (Fig. 6). Altogether, BBR’s impacts on salt bridges of MdfA were concentrated at the cytoplasmic side for both inward and outward conformations. Reduction of interaction strength in MdfA^inward^ may confine the driving force for its transition back to the ground state, and the moderately enhanced ionic interactions of MdfA^outward^ may increase the energy for its activation.

### BBR altered MdfA’s interaction with water and membrane.

**FIG 7:**
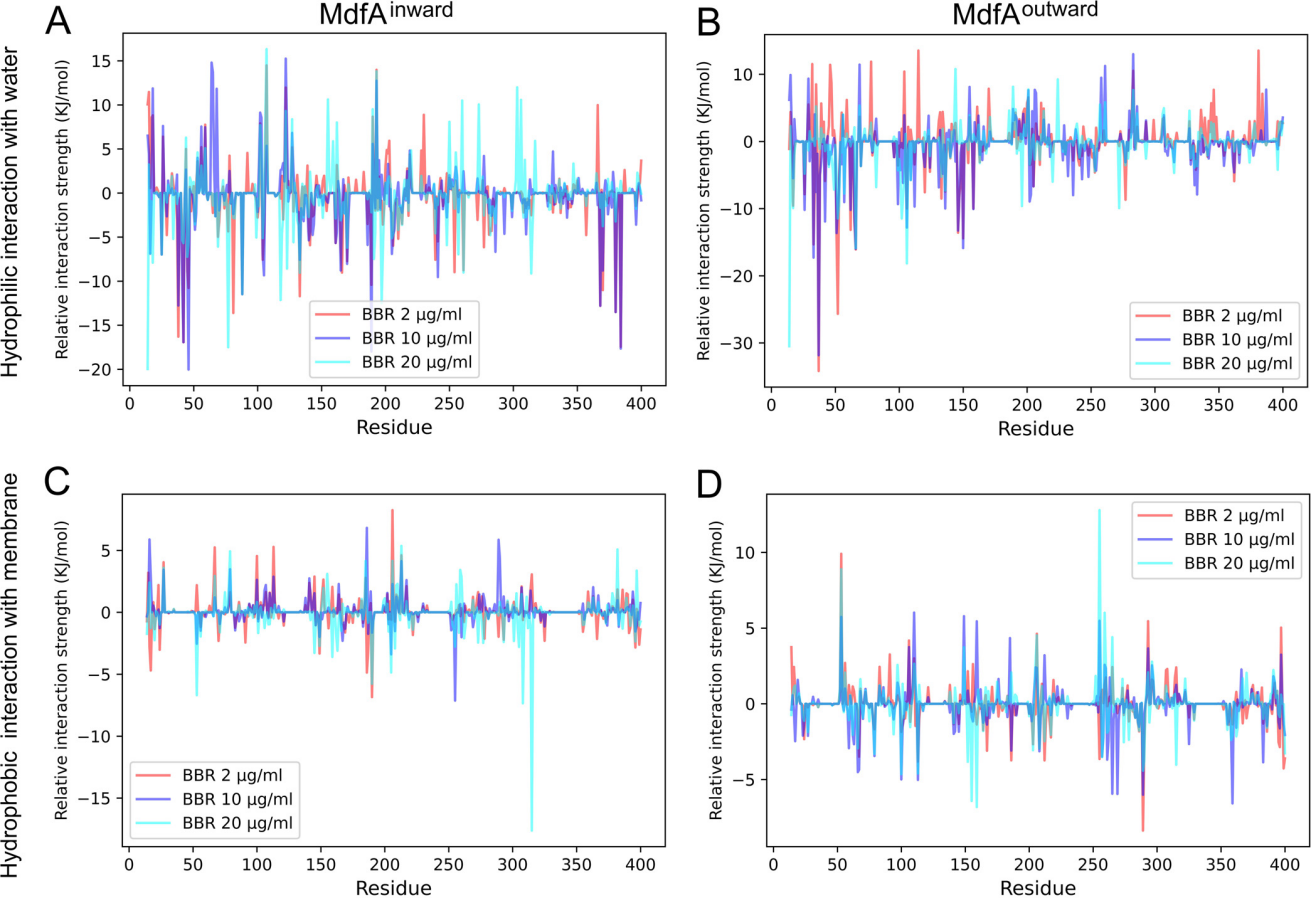
BBR affected MdfA’s hydrophilic interaction with water and the hydrophobic interaction with membrane. Average interaction strength of each residue was calculated throughout the simulations. Relative interaction strength (strength of each residue minus that of the control) was used to illustrate the differences as BBR was added.

MdfA’s interactions with water and membranes provide additional driving forces for the conformational transition due to their fluidity (8). Therefore, we calculated the average interaction strengths throughout the simulations (Fig. 7). For MdfA^inward^, its overall hydrogen bond with water was reduced by nearly 0.35 kJ/mol when BBR was added. The regions that sharply decreased interactions with water were located near Gln14, Gln46, Arg198, and Gly376. Interestingly, these data were in accordance with the hydrophobic interactions between MdfA and BBR (Fig. 4), indicating that BBR reduced MdfA’s interactions with water. This phenomenon was more obvious for MdfA^outward^. The overall hydrogen bond energy between MdfA and water was decreased by 0.6 kJ/mol. Notably, the most significant decrease was concentrated from Leu41 to Val44, the area of which also had elevated hydrophobic interaction with BBR (Fig. 4).

In contrast, the overall hydrophobic interactions between MdfA and membrane were rarely changed as BBR was added. The alterations of hydrophobic interactions were concentrated in specific residues, since we did not observe the entrance of BBR molecules into the central channel. For MdfA^inward^, significantly reduced interaction strengths were found at Ile308 and Trp315, two residues at the periplasmic side of the C-repeat. In contrast, for MdfA^outward^, sharply enhanced interactions were observed at Ile51 and Glu256, which were also at the periplasmic side. Nevertheless, decreased interactions were detected at Leu156 and Ile289, which were located in the middles of helices. Together with the data of hydrophobic interactions between BBR and MdfA, residues located at the periplasmic side that interacted with BBR also had elevated hydrophobic interactions with membranes. BBR molecules were also fluid in the simulation system, while most of the BBR molecules formed stable interactions with certain residues at each side of MdfA. Therefore, we can conclude that BBR altered the areas of MdfA’s interaction with membranes, while the total interaction strength was rarely affected.

## DISCUSSION

Efflux pumps in Gram-negative pathogens contribute to elevated level of antibiotic resistance. With the increased frequency of horizontal transfer of efflux pumps, there is an urgent need to discover safe and efficient efflux pump inhibitors. Plant secondary metabolites have been demonstrated as an ideal source of efflux pump inhibitors (22). Among these compounds, BBR is a promising one, due to its safety for humans, as it has long been used to deal with intestinal infection (41). Even though the MICs of BBR against Gram-negative pathogens are usually much higher than expected (42), it is still a useful adjuvant drug as an efflux pump inhibitor. Herein, we demonstrated that BBR can inhibit the activity of MdfA, an MFS efflux pump in E. coli that can confer ciprofloxacin resistance. By using molecular biology approaches, we indicated that BBR increased intracellular ciprofloxacin concentration to a meaningful level and restored ciprofloxacin susceptibility of the reporter strain. Moreover, we used molecular dynamics simulation to investigate the effect of BBR on the important factors for MdfA’s conformational transition. In summary, we provided strong evidence for the extended application of BBR in antibiotic treatment of bacterial infections.

When used to deal with intestinal infection, low levels of BBR can disrupt bacterial fimbriae biosynthesis and reduce the adherence of pathogens to the inner surface of the intestine (23, 43). Indeed, except for its effect on fimbriae, BBR’s antibacterial mechanisms have been demonstrated to affect several targets, such as cell division, substrate transport, DNA replication and transcription, carbon metabolism, and energy production (44–47). However, an antibacterial effect of BBR is quite inefficient for its use as an antibiotic. For example, BBR’s MIC against E. coli is higher than 500 μg/mL, and the MIC is even higher against other clinical pathogens (42, 48, 49). Conversely, low levels of BBR can exhibit inhibitory activity against efflux pumps and restore antibiotic susceptibility. BBR has been verified to affect Mdr1p of the MFS family at 32 μg/mL and MexXY-OprM and AcrAB-TolC of the RND family at 64 μg/mL (25, 50). As an ortholog of Mdr1p from C. albicans, MdfA has similar structure and can also be inhibited by BBR at 20 μg/mL. Therefore, these data provide strong evidence for BBR’s application as an inhibitor of MFS efflux pumps and which confers quinolone resistance of human pathogens.

Even though the role of MFS efflux pumps in regulating antibiotic resistance is clear, discovery and synthesis inhibitors are still limited by the unanswered export mechanism. Drug efflux is a dynamic process accompanied with large conformational transition. However, current computational resources are still difficult to describe as the whole cycle of conformational transition in a conventional molecular dynamics simulation, although the inward and outward structures of MdfA have been captured. According to previous research from ours and the other groups, driving forces of MFS efflux pumps are deduced to be composed of ionic interactions between N- and C-repeats, with hydrophilic and hydrophobic interactions with water and membrane (8, 51). Interestingly, most of the salt bridges have been found to be located at the cytoplasmic side, even for the inward conformation. The activation of MdfA can be concluded as follows. At the ground state of MdfA with the outward conformation, protonation of key residues (e.g., Asp34) in the mouth of the opening side initiates the conformational transition of MdfA (14). With the fluidity of water and membrane, the periplasmic side of the N-repeat moves close to the C-repeat. At the same time, the amount of salt bridges at the cytoplasmic side is increased transiently (Fig. 5) and energies are stored as strong ionic interactions at the mouth of the cytoplasmic side. After accommodation of substrate from the cytoplasm, opposite movement was initiated by deprotonation, and the strong ionic interactions at the cytoplasmic side drive the transition back to MdfA’s ground state.

According to this hypothesis, BBR’s impacts on the conformational transition of MdfA can be classified into two major parts. First, BBR disrupted the salt bridges as it aggregated to the periplasmic and cytoplasmic surfaces of MdfA. Activation of MdfA depended on a sharply decreased interaction strength of Glu136-Arg336, while for MdfA^outward^, supply of BBR resulted in elevated interactions of this salt bridge. On the other side, an altered salt bridge of Glu136-Lys346 was important for energy storage for MdfA^inward^. Similarly, ionic interaction possibilities of this salt bridge were gradually reduced with increased BBR concentrations. Second, MdfA’s hydrophilic interaction with water is also decreased, especially at the ground state (Fig. 6B). Our previous work demonstrated that hydrophilic interactions with water at the periplasmic side are crucial for KmrA’s activity, which is an MdfA ortholog in K. pneumoniae that can also confer ciprofloxacin resistance (51, 52). Therefore, aggregations of BBR to the surface of MdfA lower its interactions with water, which subsequently reduces the driving force of activation.

When 20 μg/mL of BBR was provided, the intracellular ciprofloxacin concentration in the recombinant E. coli was nearly 70% compared to that of the experiments with 50 μg/mL verapamil. These results indicated that BBR is a promising MdfA inhibitor for clinical use. However, when the concentration of BBR was elevated to 50 μg/mL, the intracellular ciprofloxacin concentration was not additionally increased. We deduced that this was caused by the self-aggregation of BBR molecules, which was also observed previously in BBR’s antifungal application (53). Therefore, formulation design and structural modification of BBR are anticipated to further increase BBR’s efficiency as an efflux pump inhibitor.

### Conclusion.

Infections caused by Gram-negative pathogens are usually difficult to manage due to drug export by efflux pumps. We demonstrated that BBR, an isoquinoline alkaloid which can be found in many plants, is a natural inhibitor of the MFS efflux pump MdfA from E. coli. By using molecular biology approaches, we determined that a low level of BBR can increase the intracellular ciprofloxacin concentration and restore the susceptibility of E. coli. We use molecular dynamics and provided insights to the mechanisms of BBR’s effect on the conformational transition MdfA. Our work facilitates the development of MFS efflux pump inhibitors.

## MATERIALS AND METHODS

### Chemicals and strains.

The E. coli strain used in this work is a standard DH5α strain which is commonly used in genetic engineering. The antibiotics tetracycline, streptomycin, roxithromycin, and nalidixic acid were formulated as 2-mg/mL stock solutions in water. Rifampin (Macklin, Beijing) was formulated as a 2-mg/mL stock in dimethyl sulfoxide (DMSO). Azithromycin and chloramphenicol (Macklin, Beijing) were formulated as 2-mg/mL stock solutions in ethanol. Ciprofloxacin (Sigma, Shanghai) was dissolved in 0.15 M sodium hydroxide at 1 mg/mL, and it was neutralized before addition to the medium. Cyanide-*m*-chlorophenylhydrazone (CCCP; Sigma, Shanghai) was dissolved in DMSO to reach a final concentration of 5 mg/mL, and 20 μg/mL of CCCP was used in the medium to inhibit the activity of efflux pumps (10, 54). The MFS-specific inhibitor verapamil (Sigma, Shanghai) was formulated at 5 mg/mL in water. Berberine (Sigma, Shanghai) was dissolved in water (1 mg/mL) or DMSO (10 mg/mL) for different applications. LB medium (containing per liter, 10 g NaCl, 10 g peptone, 5 g yeast extract, 15 g agar if necessary; Bioroyee, Beijing) was prepared as broth according to the manufacturer’s instructions. All strains were grown overnight in a 37°C shaking incubator (200 rpm) prior to initiating the experiment.

### Genetic modifications.

The backbone of expression vector pET-28a was used for overexpression of *mdfA*. Since the major products under the T7 promoter are inclusion bodies when cultivated at 37°C, this was thereby replaced by *tac* promoter (55). Moreover, the chloramphenicol resistance gene from pCP20 was amplified and used to replace the kanamycin resistance gene, because kanamycin was used for gene deletion. Gene deletion was conducted via the allelic exchange method with a pmob-sacB plasmid (56). Briefly, 1,000 bp of the upstream and downstream regions of the desired gene were amplified and then assembled with linear pmob-sacB plasmid through the Gibson assembly method. The recombinant plasmid was transformed into E. coli, and kanamycin (10 μg/mL) was used to sustain the plasmid. After cultivation, bacteria were spread on an LB plate with kanamycin, and genomic insertion of the kanamycin resistance gene was identified by colony PCR. Next, recombinants were cultivated in LB medium in the presence of 10% sucrose to eliminate the kanamycin resistance gene. A single colony after PCR verification of the gene deletion was subjected to further study. Primers used in this study are listed in Table S1 in the supplemental material.

### Determination of MICs.

MICs were determined by use of a microplate assay to test antibiotic activity against the recombinant strains (57). The medium used in overnight culture and MIC assays was LB broth containing chloramphenicol (100 μg/mL) and the inducer IPTG (0.5 mM). First, the optical density at 600 nm (OD_600_) of the overnight culture was measured to maintain equal amount of cells in each group, and cells were diluted 10,000-fold into a 96-well plate. Then, 2-fold dilutions of each working solution were prepared, and the last cell in each row was set as a control. Plates were incubated at 37°C for 12 h. The experiments were conducted for more than three biological replicates.

### Growth curve.

Strains were grown overnight in LB medium containing chloramphenicol (100 μg/mL) and the inducer IPTG (0.5 mM). Cells were diluted 10,000-fold into the new LB medium from an overnight culture and transferred into a 96-well plate for growth curve evaluation. The efflux pump inhibitor verapamil was added into the medium when necessary to reach a final concentration of 50 μg/mL (58, 59). Bacterial growth was automatically monitored in a SpectraMax i3x plate reader at 37°C and with 100 μL medium in each cell of the 96-well plate, and the data were recorded every 20 min for 24 h. Three biological replicates were conducted for the experiment.

### Determination of intracellular ciprofloxacin concentrations.

The intracellular ciprofloxacin concentrations of the recombinant strains were determined according to methods described previously (60, 61). Briefly, recombinant strains were cultured in LB broth overnight. Cultures were diluted 100-fold in fresh LB medium and then cultivated for 2 to 3 h to the logarithmic phase. Cells were then harvested by centrifugation, washed with phosphate-buffered saline (PBS) buffer (50 mM, pH 7.0), and resuspended in fresh LB medium at 37°C to an OD_600_ of 1.0. Subsequently, 1 mL of sample was used for determination of intracellular ciprofloxacin, and 5 μg/mL ciprofloxacin was supplied in the medium. After treatment with ciprofloxacin for 30 min, the sample was evenly divided into two parts. The first half of the sample was used to measure cell dry weight at the end of experiment, and the other half was used to determine ciprofloxacin concentration. Samples were immediately diluted with 500 μL cold PBS buffer. After centrifugation, cells were washed twice with cold PBS buffer and then resuspended in 0.1 M glycerin hydrochloride (pH 3.0) and shaken at 37°C overnight. After centrifugation, the supernatants were subjected to high-performance liquid chromatography (HLPC) analysis after filtration through a 0.22-μm filter. HPLC analysis was conducted in a Shimadzu LC-16 system equipped with a C_18_ column and a UV detector. The liquid phase was water-methanol-phosphate acid at the ratio of 60/40/0.01. Flow rate was set at 1.0 mL/min, and the detective wavelength was set at 280 nm.

### Structures and software.

The inward (4zp0) and outward (6gv1) structures of MdfA were downloaded from the RCS PDB databank followed by deleting ligands (62). After comparing the amino acid sequences derived from their PDB structures, one residue substitution was discovered between the inward and outward structure of MdfA (131Q for outward and 131R for inward). To make the simulations comparable, we made the swap of R131Q of the MdfA^inward^ structure followed by energy minimization. Then, the two structures were applied as the initial states of the two simulations. Structural modification and molecular dynamics simulations were conducted in the commercial Yasara Structure software suite (version 21.6.17), for which we have the license.

### Molecular dynamics simulation.

All the molecular dynamics simulations were carried out in Yasara Structure using the Amber 14 force field, and the periodic boundary condition was used. The macro md_run_membrane provided by Yasara Structure was applied for all the simulations. First, a membrane structure was generated and compressed to prevent the entrance of water molecules in a simulation box. Then, the structure of MdfA was inserted into the membrane in a square simulation cell with a length of 90 Å. Next, BBR molecules were added into the system to reach final concentrations equal to those in the wet experiments, and water molecules were filled into the simulation box. In the simulation box, several Na^+^ cations were also added to neutralize the system. An energy minimization was performed to eliminate improper contacts in each system. The steepest descent method was used in the first 5,000 steps, and the conjugate gradient method was applied in the last 5,000 steps. After the energy minimization, each system was heated gradually from 0 K to 310 K. Each system was then equilibrated for 500 ps at constant temperature (310 K) and pressure (10^5^ Pa) conditions via Langevin dynamics (collision frequency of 1.0 ps^−1^). Each simulation was performed for 200 ns at 310 K and 10^5^ Pa. The snapshot was recorded every 0.1 ns.

### Statistical analysis.

Analysis of the trajectory of simulation was also conducted in Yasara according to the manufacturer’s instructions. RMSD and RMSF were used to evaluate the total and local flexibilities, respectively. Ionic interaction (salt bridge) was identified if the distance between positively and negatively charged residues was lower than 5 Å and the interaction strength was calculated. Hydrophobic interaction strength, defined using Yasara Structure, was used to determine the hydrophobic interaction between MdfA and membrane. All the plots were made in the Python Matplotlib module, and the structures were visualized in PyMOL. Data for growth curves are presented as means ± standard errors of means (SEM). Statistical significances are indicated by *P* values determined by one-way analysis of variance (ANOVA).
