# Supplementary material for: Role of Berberine as a Potential Efflux Pump Inhibitor against MdfA from Escherichia coli: In Vitro and In Silico Studies
Source: Microbiol Spectr. 2023 Feb 14;11(2):e03324-22. doi: 10.1128/spectrum.03324-22 (PMC10100983; doi:10.1128/spectrum.03324-22)
Supplement: Supplemental file 1 — Table S1 and Fig. S1 to S3. Download spectrum.03324-22-s0001.pdf, PDF file, 0.5 MB [file spectrum.03324-22-s0001.pdf]

Supplemental Table 1. Primers used in this work.

Name	Sequence
mdfa-F	5'-tagaaataattttgtttaactttaagaaggagatatacccatgcaaaataaattagcttc-3'
mdfa-R	5'-ggccccaaggggttatgctagttattgctcagcggtgcccttacccttcgtgagaatttc-3'
mdfa-18sac-1F	5'-ccaagcttgcatgcctgcaggtcgactctagaggatccccgcaaagatgtttttaatggc-3'
mdfa-18sac-1R	5'-gattattattggcgaagaaattgcaaaaaaatgcctgattgctttgtgcg-3'
mdfa-18sac-2F	5'-cgcacaaagcaatcaggcatttttttgcaatttcttcgccaataataatc-3'
mdfa-18sac-2R	5'-aacagctatgaccatgattacgaattcgagctcggtacccttatcgctgcgttttaattc-3'
mdfa-rhab-1F	5'-gctcaaattggaatcaggtttgtgccaataccagtagcccgcaaagatgtttttaatggc-3'
mdfa-check-F	5'-cctgatcgcacaaagcaatcaggca-3'
mdfa-check-R	5'-ttaaactctgcgcgattattattgg-3'

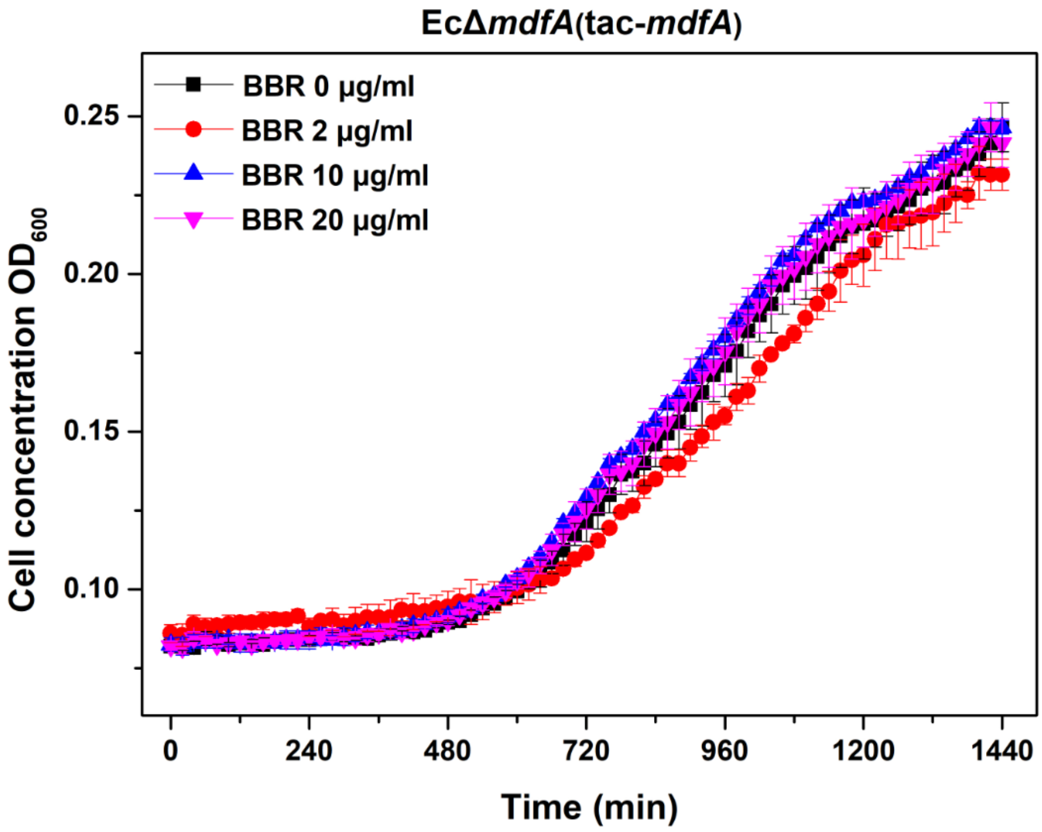

Figure S1. Low levels of BBR had no effect on cell growth of the reporter strain. Cell growth curve was obtained from a plate reader. All the experiments were conducted with at least three biological replicates. Data were presented at mean values ± standard error.

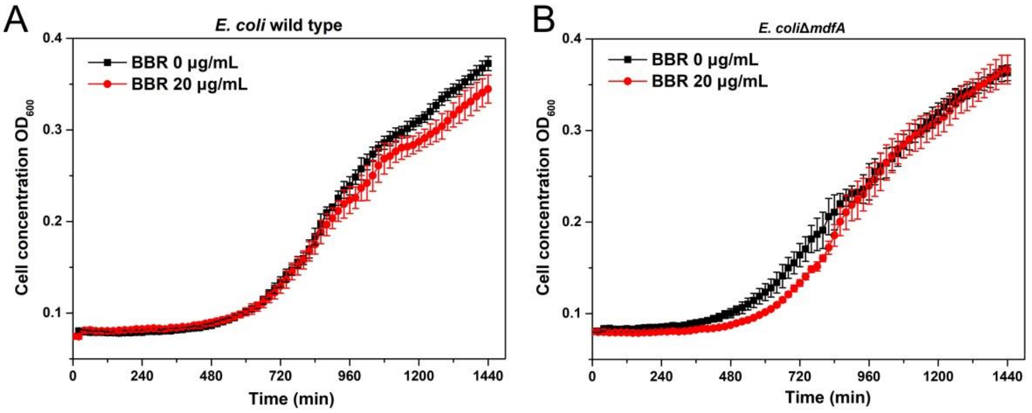

Figure S2. Low levels of BBR had no effect on cell growth of the wild type and the mdfA mutation strains. Cell growth curve was obtained from a plate reader. All the experiments were conducted with at least three biological replicates. Data were presented at mean values ± standard error.

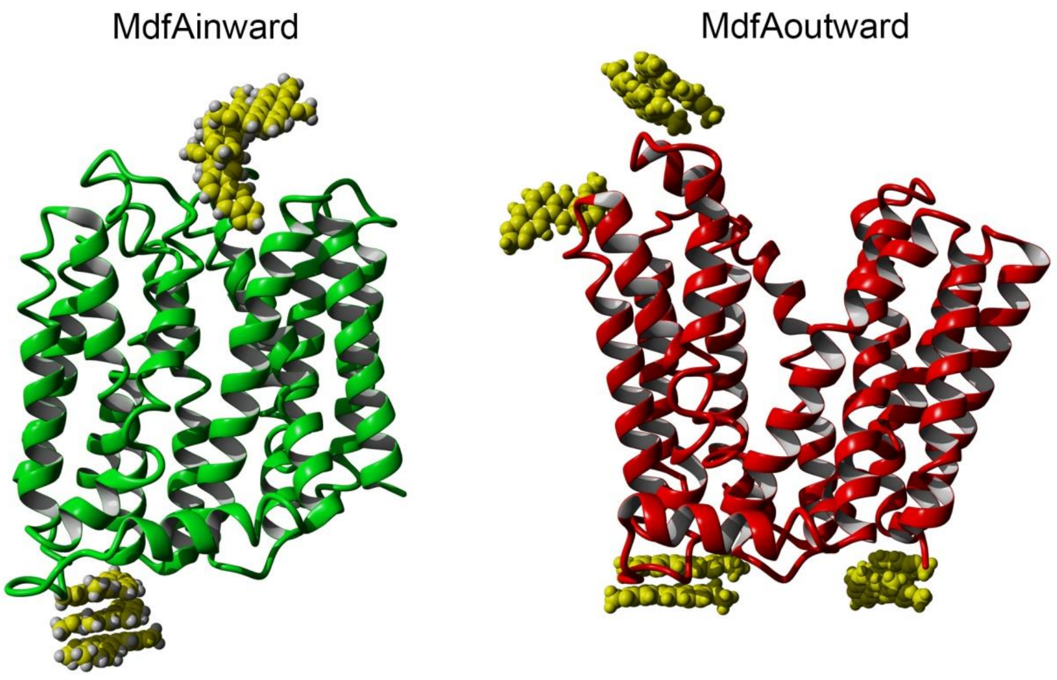

Figure S3. Visualization the interactions of MdfA^{inward} and MdfA^{outward} with BBR molecules in the simulation. Molecules colored yellow represent BBR.
